# Triggers, Inhibitors, Mechanisms, and Significance of Eryptosis: The Suicidal Erythrocyte Death

**DOI:** 10.1155/2015/513518

**Published:** 2015-03-04

**Authors:** Elisabeth Lang, Florian Lang

**Affiliations:** Department of Physiology, University of Tübingen, Gmelinstrasse 5, 72076 Tübingen, Germany

## Abstract

Suicidal erythrocyte death or eryptosis is characterized by erythrocyte shrinkage, cell membrane blebbing, and cell membrane scrambling with phosphatidylserine translocation to the erythrocyte surface. Triggers of eryptosis include Ca^2+^ entry, ceramide formation, stimulation of caspases, calpain activation, energy depletion, oxidative stress, and dysregulation of several kinases. Eryptosis is triggered by a wide variety of xenobiotics. It is inhibited by several xenobiotics and endogenous molecules including NO and erythropoietin. The susceptibility of erythrocytes to eryptosis increases with erythrocyte age. Phosphatidylserine exposing erythrocytes adhere to the vascular wall by binding to endothelial CXC-Motiv-Chemokin-16/Scavenger-receptor for phosphatidylserine and oxidized low density lipoprotein (CXCL16). Phosphatidylserine exposing erythrocytes are further engulfed by phagocytosing cells and are thus rapidly cleared from circulating blood. Eryptosis eliminates infected or defective erythrocytes thus counteracting parasitemia in malaria and preventing detrimental hemolysis of defective cells. Excessive eryptosis, however, may lead to anemia and may interfere with microcirculation. Enhanced eryptosis contributes to the pathophysiology of several clinical disorders including metabolic syndrome and diabetes, malignancy, cardiac and renal insufficiency, hemolytic uremic syndrome, sepsis, mycoplasma infection, malaria, iron deficiency, sickle cell anemia, thalassemia, glucose 6-phosphate dehydrogenase deficiency, and Wilson's disease. Facilitating or inhibiting eryptosis may be a therapeutic option in those disorders.

## 1. Introduction

The lifespan of circulating erythrocytes is limited by senescence to 100–120 days [1–3]. In senescent erythrocytes hemichromes bind to and cluster the anion exchanger protein band 3 (AE1), leading to attachment of complement C3 fragments and antiband 3 immunoglobulins [4]. Prior to senescence, erythrocytes may enter suicidal death or eryptosis, characterized by erythrocyte shrinkage and cell membrane scrambling with translocation of phosphatidylserine from the inner leaflet of the cell membrane to the erythrocyte surface [5, 6]. Phosphatidylserine avidly binds annexin V, which is thus employed to identify eryptotic cells [5, 6].

The present paper lists triggers and inhibitors or eryptosis, the mechanisms involved in the regulation of eryptosis, and the (patho-) physiological significance of eryptosis. The reader is encouraged to study earlier reviews on further aspects of eryptosis [6–12].

## 2. Triggers and Inhibitors of Eryptosis

As listed in Table 1, a wide variety of xenobiotics and endogenous small molecules may trigger eryptosis. Moreover, eryptosis is triggered by several other stressors, such as osmotic shock [13], energy depletion [14], oxidative stress [11, 15], or increase of temperature [16]. Eryptosis is inhibited by a variety of xenobiotics (Table 2), by nitric oxide [17], and by erythropoietin [18, 19].

The susceptibility to stimulation of eryptosis increases with erythrocyte age [20]. The enhanced spontaneous eryptosis of aged erythrocytes is abrogated by the antioxidant N-acetyl-L-cysteine [20]. The mechanism rendering aged erythrocytes particularly vulnerable to eryptosis remained ill-defined [20]. Young erythrocytes are particularly sensitive to suicidal death following decline of erythropoietin, a phenomenon termed neocytolysis [21].

Erythrocytes from newborns are relatively resistant to several triggers of eryptosis but are highly susceptible to eryptosis following oxidative stress [22–24]. The exquisite sensibility of fetal erythrocytes to oxidative stress is presumably instrumental for their removal following birth. The high oxygen affinity of fetal hemoglobin is favourable in the oxygen-depleted intrauterine environment but not after birth [25]. Thus, replacement of fetal erythrocytes by adult erythrocytes is mandatory for adequate oxygen transport after birth.

## 3. Signaling Regulating Eryptosis

A major trigger of eryptosis is the increase of cytosolic Ca^2+^ activity ([Ca^2+^]_i_) [6]. The increase of [Ca^2+^]_i_ mainly results from Ca^2+^ entry through Ca^2+^-permeable unselective cation channels [26, 27], which are permeable to both Na^+^ and Ca^2+^ [28]. The channels are activated by prostaglandin E_2_ (PGE_2_) [29, 30]. Pharmacological inhibition of cyclooxygenase or phospholipase-A_2_ disrupts the activation of the channels following osmotic shock [29]. The Ca^2+^ permeable unselective cation channels are further activated by isosmotic replacement of NaCl with sorbitol [28] and by substitution of extracellular Cl^−^ with gluconate, Br^−^, I^−^, or SCN^−^ [28]. They are further activated by oxidative stress or defects of antioxidative defence [31–34], which thus trigger Ca^2+^ entry and eryptosis [5, 28]. Activation of the channels by oxidative stress is reversed by the reducing agent dithiothreitol [5]. Accordingly, erythrocytes are protected against oxidative stress by several antioxidants including vitamin E [35–37] glutathione [38], salidroside [39], 7-monohydroxyethylrutoside [40], trolox [31], or N-acetylcysteine [18, 20]. The channels involve the transient receptor potential channel TRPC6 [26]. The increase in [Ca^2+^]_i_ and eryptosis following Cl^−^ removal were thus blunted in erythrocytes from gene-targeted mice lacking TRPC6 [26].

The increase of [Ca^2+^]_i_ following activation of the cation channels is followed by cell shrinkage due to activation of Ca^2+^-sensitive K^+^ channels [41, 42], cell membrane hyperpolarization, increase in the electrical driving force for Cl^−^ exit, and the cellular loss of KCl with osmotically obliged water [43]. The Cl^−^ exit requires erythrocyte Cl^−^ channels [44], which are activated by oxidative stress [45, 46]. Cell shrinkage occurs as long as cellular K^+^ loss through the Ca^2+^-sensitive K^+^ channels outcasts the Na^+^ entry through the unselective cation channels. Sustained exit of K^+^ and entry of Na^+^ may lead to dissipation of respective ion gradients across the cell membrane, to cell membrane depolarization, and thus to entry of Cl^−^ and cell swelling [47]. Excessive cell swelling jeopardises the integrity of the cell membrane and may trigger hemolysis [28, 47].

An increase of [Ca^2+^]_i_ further stimulates cell membrane scrambling with breakdown of phosphatidylserine asymmetry of the erythrocyte cell membrane and translocation of phosphatidylserine to the erythrocyte surface [48]. Ca^2+^ sensitivity of the machinery leading to cell membrane scrambling is increased by ceramide [6]. Osmotic shock and a variety of further stimulators of eryptosis activate a phospholipase A_2_ with following formation platelet-activating factor, which in turn activates a ceramide producing sphingomyelinase [6].

Triggering cell membrane scrambling may involve but does not necessarily require activation of caspases [6, 103, 224], which are expressed in erythrocytes [225, 226], cleave the anion exchanger AE1 [225], and stimulate phosphatidylserine exposure of erythrocytes [227]. Caspase activation participates, for instance, in the triggering of eryptosis by leukotrienes [16] and *α*-lipoic acid [50]. The caspases are further activated by oxidative stress [228]. Ca^2+^ entry and Ca^2+^-dependent cell membrane scrambling do, however, not require activation of caspases [48, 76, 229].

Signaling influencing eryptosis further involves Janus-activated kinase JAK3 [222]. The kinase is phosphorylated at Tyr 980 and thus activated by energy depletion [222]. JAK3 activation contributes to the stimulation of cell membrane scrambling following energy depletion and the effect of energy depletion on eryptosis is blunted by pharmacological or genetic knockout of JAK3 [222].

Eryptosis following energy depletion is inhibited by the energy sensing AMP-activated kinase (AMPK) [27]. Even without induction of energy depletion, eryptosis is increased in AMPK*α*1-deficient mice [27]. The excessive eryptosis in AMPK*α*1-deficient mice leads to profound anemia and splenomegaly due to trapping of eryptotic erythrocytes in the spleen [27]. AMPK deficiency is paralleled by downregulation of p21-activated kinase 2 (PAK2) which presumably participates in the inhibition of eryptosis [111].

Pharmacological evidence points to a role of casein kinase 1*α* (CK1*α*) in the increase in [Ca^2+^]_i_ and subsequent stimulation of eryptosis upon exposure of erythrocytes to oxidative stress or following energy depletion [143]. Pharmacological activation of CK1*α* opens cation channels and thus triggers Ca^2+^ influx into erythrocytes [144]. Osmotic shock activates p38 kinase in human erythrocytes [183] and pharmacological inhibition of p38 kinase blunts the eryptosis following osmotic shock [183]. Eryptosis is apparently inhibited by sorafenib- [153] and sunitinib- [157] sensitive kinases.

Eryptosis is further inhibited by cGMP-dependent protein kinase (cGKI) [217]. cGKI deficient mice suffer from severe anemia and splenomegaly due to excessive eryptosis [217]. cGKI deficiency is at least partially effective by increasing [Ca^2+^]_i_ [217]. The kinase is stimulated by nitric oxide (NO) [230–233], a powerful inhibitor of eryptosis [17]. NO is stored in erythrocytes and may be released upon deoxygenation of hemoglobin [234–236]. Eryptosis is inhibited by NO-donors such as nitroprusside [17] at concentrations within or even below the range of those effective in nucleated cells [237, 238]. NO is at least partially effective downstream of Ca^2+^ as it protects against eryptosis induced by the Ca^2+^ ionophore ionomycin without appreciably affecting the ionomycin-induced increase of [Ca^2+^]_i_. NO blunts apoptosis of nucleated cells in part by caspase inhibition [239, 240]. However, caspases are not required for the stimulation of eryptosis following increase of [Ca^2+^]_i_ [6]. Similar to its effect in nucleated cells [241–245] NO increases nitrosylation of enzymes, which are necessary for induction of cell membrane scrambling [17]. Conversely, protein S-nitrosylation is decreased by treatment of erythrocytes with ionomycin. Enzymes affected include the antiapoptotic enzyme thioredoxin, which is activated by S-nitrosylation [17, 242]. As shown in nucleated cells compromised thioredoxin activity enhances oxidative stress [242, 243]. The effect of NO is partially mimicked by dibutyryl-cGMP [17]. In contrast to low concentrations [17], excessive concentrations of the nitroprusside stimulate eryptosis presumably through oxidative stress [246–248]. NO release is particularly fast from HbF, which has thus a particular potency to counteract eryptosis and inducing vasodilation [249, 250]. In sickle cell disease increased levels of antisickling HbF counteract oxidative stress [251] and presumably eryptosis.

Collectively erythrocyte survival and eryptosis are regulated by an amazingly complex cellular machinery involving [Ca^2+^]_i_, ceramide, oxidative stress, caspases, nitroxide, and a variety of kinases. Most triggers of eryptosis are mainly effective by increasing [Ca^2+^]_i_ and/or enhancing ceramide abundance in the cell membrane. Unlike in apoptosis of nucleated cells, caspases do not play a dominant role in the triggering of eryptosis. Survival of erythrocytes does require the activity of several kinases including AMPK and cGKI. Activation of other kinases, such as CK1*α* and JAK3, triggers eryptosis. The phosphorylation targets of the kinases required for the stimulation or inhibition of eryptosis are still ill-defined. Clearly, tremendous additional experimental effort is required for full understanding of the eryptotic machinery.

## 4. Significance of Eryptosis

Phosphatidylserine exposing erythrocytes are rapidly cleared from circulating blood [190] as phosphatidylserine binds to respective receptors of phagocytosing cells leading to engulfment and degradation of the affected erythrocytes [6]. As long as accelerated loss of eryptotic erythrocytes is matched by an equivalent increase of erythropoiesis, the number of circulating erythrocytes remains unaffected [6]. The enhanced turnover of erythrocytes is then reflected by an increased percentage of reticulocytes [6]. As soon as the loss of erythrocytes by eryptosis outcasts the formation of new erythrocytes, anemia develops [6].

Phosphatidylserine-exposing erythrocytes further adhere to the vascular wall by binding of phosphatidylserine to endothelial CXC-Motiv-Chemokin-16/Scavenger receptor for phosphatidylserine and oxidized low density lipoprotein (CXCL16/SR-PSOX) [252]. Further structures binding phosphatidylserine-exposing erythrocytes include the heparin-binding domain [253] of endothelial or subendothelial thrombospondin-1 (TSP) [254] or endothelial phosphatidylserine receptors [255]. As adherence of eryptotic erythrocytes to endothelial cells is virtually abrogated by silencing of endothelial CXCL16/SRPSO or by coating phosphatidylserine at the erythrocyte surface with annexin V, the erythrocytes bind apparently in large part by interaction of phosphatidylserine with endothelial CXCL16/SRPSO [252]. Phosphatidylserine exposing erythrocytes further adhere to blood platelets [252, 256]. The adherence of phosphatidylserine-exposing erythrocytes to vascular wall and to blood platelets compromises microcirculation [252, 257]. Phosphatidylserine-exposing erythrocytes may further trigger blood clotting and thus foster thrombosis [257].

## 5. Diseases Associated with Enhanced Eryptosis

Increased eryptosis contributes to the pathophysiology of diverse clinical disorders (Table 3) and is observed in a variety of knockout mice (Table 4). Eryptosis is augmented following dehydration, an effect paralleled by increase of 1,25(OH)_2_D_3_ plasma levels [188]. Along those lines, enhanced eryptosis is observed in Klotho deficient mice which suffer from excessive 1,25(OH)_2_D_3_ formation [218, 219]. The eryptosis in those mice is blunted by vitamin D deficient diet [218, 219]. Eryptosis is further triggered by severe phosphate depletion [193], an effect presumably due to compromised ATP generation.

The percentage of phosphatidylserine-exposing erythrocytes is enhanced in iron deficiency which leads to decrease of cell volume and increase of cytosolic Ca^2+^ concentration [190]. The enhanced [Ca^2+^]_i_ results from activation of the Ca^2+^ permeable unselective cation channels [190] and possibly from increased oxidative stress [258]. The enhanced eryptosis is paralleled by accelerated clearance of iron-deficient erythrocytes, which thus compounds the anemia [190].

Eryptosis is enhanced in malignancy [259]. Little is known about underlying mechanisms. The effect is compounded by cytostatic treatment, as a wide variety of cytostatic drugs do not only trigger apoptosis of tumor cells but as well suicidal death of erythrocytes (Table 1).

The percentage of phosphatidylserine-exposing erythrocytes in circulating blood is increased in diabetic patients [120, 224, 260]. Eryptosis is stimulated by methylglyoxal [120], which accumulates in hyperglycemia [261]. Methylglyoxal is at least partially effective by interference with glycolysis and by decrease of ATP and GSH concentrations [120]. Hyperglycemia imposes oxidative stress [262] with GSH depletion [262], increase of malondialdehyde concentrations [262], increased SOD activity [263], and erythrocyte lipid peroxidation [260]. Eryptosis has further been postulated to be enhanced in metabolic syndrome [191].

Eryptosis is further enhanced in chronic kidney disease (CKD) [19, 196], a condition invariably associated with anemia [264, 265]. The anemia of CKD is commonly attributed to lack of renal erythropoietin release and subsequent impairment of erythropoiesis [266, 267]. CKD is further commonly paralleled by iron deficiency [266, 268–271]. However, CKD leads to enhanced percentage of phosphatidylserine exposing erythrocytes [19, 196, 197] and accelerated clearance of circulating erythrocytes [272]. Accordingly, profound anemia occurs even in patients who are adequately treated with erythropoietin and thus have normal reticulocyte numbers in circulating blood [197]. Erythrocytes from CKD patients are exposed to oxidative stress [273]. The enhanced eryptosis in CKD patients is further partially due to hyperphosphatemia [139] and in part due to accumulation of uremic toxins, such as vanadate [167], acrolein [49], indoxyl sulfate [110], and methylglyoxal [120]. Acrolein and methylglyoxal may be partially effective by depleting the cells from glutathione [274].

Eryptosis is triggered by hemolytic uremic syndrome (HUS), characterized by hemolytic anemia with fragmented erythrocytes, thrombocytopenia, and acute renal failure [198]. The disorder may result from intoxication with bacterial shiga toxin or from complement activation due to lack of complement-inactivating factor H [198]. Plasma from HUS patients triggers in erythrocytes from healthy volunteers phosphatidylserine exposure, cell shrinkage, increase in cytosolic Ca^2+^ activity, and ceramide formation [198]. The effect of patient plasma on eryptosis is abolished by plasmapheresis or filtration at 30 kDa. Eryptosis is similarly triggered by activated complement [198]. Mechanisms involved include oxidative stress and lipid peroxidation [275]. Both are imposed by neutrophils [276].

Severe eryptosis is observed in sepsis [199]. Again, plasma from septic patients stimulates phosphatidylserine exposure, cell shrinkage, increase in cytosolic Ca^2+^ activity, and ceramide formation in erythrocytes from healthy individuals [199]. The effect is mimicked by exposure of erythrocytes to supernatant of pathogens and paralleled by enhanced sphingomyelinase activity [199]. Again, sepsis imposes oxidative stress [277], which may participate in the triggering of eryptosis.

Enhanced eryptosis is observed in Wilson's disease [81], a genetic disorder caused by Cu^2+^ accumulation due to inactivating mutations of Cu^2+^-secreting ATP7B [278]. The eryptosis is paralleled by mild anemia [278]. Eryptosis in Wilson's disease is in large part secondary to activation of acid sphingomyelinase with ceramide formation [81]. Moreover, erythrocytes from patients with Wilson's disease are exposed to oxidative stress [279] presumably due to copper-related oxidants [280].

A mutation of the anion exchanger AE1 in humans [214] or genetic knockout of the carrier in mice [221] is followed by opening of the Ca^2+^ permeable unselective cation channels in erythrocytes and thus by accelerated eryptosis. An extremely rare mutation of GLUT1 turns the carrier into a Ca^2+^ permeable unselective cation channel similarly enhancing eryptosis [215].

The susceptibility to eryptosis is increased in sickle cell anemia, thalassemia, and glucose 6-phosphate dehydrogenase deficiency [203, 281, 282]. Enhanced phosphatidylserine exposure fosters adhesion of the erythrocytes to endothelial cells [283–286]. Adhesion of sickle cells to the pulmonary vascular wall is fostered by activated neutrophils [283]. The binding of sickle cells to endothelial cells is decreased by annexin V indicating that it is largely due to endothelial adhesion of erythrocytic phosphatidylserine [287] but may, in addition, involve very late-activating antigen-4 (VLA-4) and CD36 [288]. HbF counteracts eryptosis and endothelial adhesion of sickle cells to endothelial cells [24, 251, 287, 289, 290]. Expression of HbF could be enhanced by hydroxyurea, which thus decreases sickling and thus vasoocclusive complications [251, 291]. HbF may, however, sensitize erythrocytes to oxidative stress-induced eryptosis (see Section 2), which may, at least in theory, limit the therapeutic benefit of hydroxyurea. Heterozygous carriers of the genetic disorders, such as heterozygous sickle cell carriers (HbA/S), do not spontaneously become suicidal and the respective individuals are virtually healthy [281]. Nevertheless, the erythrocytes are more sensitive to the eryptotic effects of oxidative stress [281].

The malaria pathogen* Plasmodium falciparum* imposes oxidative stress on the host erythrocytes and thus activates several ion channels in the erythrocyte cell membrane [292], including the oxidant-sensitive Ca^2+^-permeable erythrocyte cation channels [45, 46, 293]. The channels accomplish uptake of nutrients, Na^+^ and Ca^2+^ as well as disposal of waste products, and are thus required for intraerythrocytic survival of* Plasmodium falciparum* [8, 281, 292–294]. The Ca^2+^ entry following activation of the Ca^2+^-permeable cation channels leads, however, to stimulation of eryptosis [32–34] and subsequent clearance of the affected erythrocytes from circulating blood [295]. The pathogen sequesters Ca^2+^ thus slowing the increase of [Ca^2+^]_i_ [296]. The pathogen further digests hemoglobin and exports the respective amino acids [297].* Plasmodium falciparum* infection eventually leads to cell membrane scrambling with exposure of phosphatidylserine [105, 294, 298, 299] and subsequent phagocytotic clearance of pathogen-containing erythrocytes [300, 301]. The pathogen may further foster erythrocyte senescence contributing to the clearance of infected cells [301, 302]. Adherence of phosphatidylserine-exposing erythrocytes to endothelial cells further leads to tissue sequestration of* Plasmodium*-infected cells allowing partial immune evasion of pathogen-containing erythrocytes [303].

Upon infection, eryptosis is accelerated in erythrocytes from heterozygous carriers of the sickle-cell trait (HbA/S), beta-thalassemia-trait, homozygous Hb-C, and G6PD-deficiency thus leading to early clearance of infected erythrocytes, decreased parasitemia, and a relatively mild course of the disease [1, 203–206, 210, 212, 281, 300]. As shown for HbA/S erythrocytes [281], spontaneous eryptosis is in those individuals usually not clinically relevant. Following infection with* P. falciparum*, however, the formation of PGE_2_, Ca^2+^ permeability, phosphatidylserine exposure at the cell surface, and removal by macrophages are all augmented in HbA/S carriers [281]. The accelerated eryptosis in iron deficiency similarly confers some protection against a severe course of malaria [304]. Moreover, parasitemia and clinical course of malaria can be favourably influenced by pharmacological stimulation of eryptosis, for example, by lead [305], chlorpromazine [85], and inhibition of NO synthase by L-NAME [201]. Importantly, the pathogen should be unable to become resistant to therapeutic acceleration of eryptosis, which depends on host cell mechanisms and is thus not at the genetic disposal of the pathogen. Along those lines, the pathogen remained unable to overcome the relative resistance of sickle cell trait carriers to malaria.

## 6. Conclusions

Similar to apoptosis of nucleated cells, eryptosis is a physiological mechanism eliminating defective erythrocytes in order to prevent hemolysis and subsequent release of hemoglobin into circulating blood. Excessive eryptosis may, however, cause anemia and impede microcirculation. Orchestration of eryptosis involves Ca^2+^-permeable unselective cation channels, ceramide, caspases, and a variety of kinases including Janus-activated kinase 3, AMP-activated kinase, cGMP-dependent protein kinase, casein kinase 1*α*, p38 kinase, protein kinase C, and p21-activated kinase 2. The sensitivity to eryptosis is enhanced in aged erythrocytes. Fetal erythrocytes are particularly sensitive to oxidative stress. Eryptosis is triggered by a wide variety of xenobiotics and enhanced eryptosis is observed in several clinical conditions including dehydration, diabetes, cardiac and renal insufficiency, hemolytic uremic syndrome, sepsis, malaria, iron deficiency, sickle cell anemia, thalassemia, glucose 6-phosphate dehydrogenase deficiency, and Wilson's disease. Drugs and nutrients inhibiting eryptosis may open novel therapeutic options in the treatment of anemia and deranged microcirculation. Eryptosis stimulating xenobiotics may at least in theory accelerate removal of* Plasmodium* infected erythrocytes and thus favourably influence the clinical course of malaria.

## Figures and Tables

**Table 1:** Stimulators of eryptosis.

Stimulators	References
A23187	[9]
Acrolein	[49]
*α*-lipoic acid	[50]
Aluminium	[18, 51]
Amantadine	[52]
Amiodarone	[53]
Amphotericin B	[54]
Amyloid	[55]
Anandamide	[56]
Anti-A IgG	[57]
Apigenin	[58]
Aristolochic acid	[59]
Arsenic	[60, 61]
Artesunate	[62]
Azathioprine	[63, 64]
Bay 11-7082	[65, 66]
Bay-Y5884	[67]
Beauvericin	[68]
Benzethonium	[69]
betulinic acid	[70]
Bismuth chloride	[71]
Cadmium	[72]
Carbon monoxide	[73]
Carmustine	[74]
Celecoxib	[75]
Ceramide (acylsphingosine)	[76]
Chlorpromazine	[77]
Chromium	[78]
Ciglitazone	[79]
Cisplatin	[80]
Copper	[81]
Cordycepin	[82]
Cryptotanshinone	[83]
Curcurmin	[84]
Cyclosporine	[85, 86]
CD95/Fas/ligand	[87]
Dermaseptin	[88]
Dicoumarol	[89]
Dimethylfumarate	[90]
Enniatin A	[91]
Estramustine	[92]
Ferutinin	[93]
Fluoxetine	[94]
FTY720	[95]
Fumagillin	[96]
Gambogic acid	[97]
Gedunin	[98]
Geldanamycin	[99]
Glycation	[100]
Glycophorin-C	[101]
Gold chloride	[102]
Gold nanorods	[103]
Gossypol	[104]
Granzyme B	[105]
Hemin	[106]
Hexavalent chromium	[107]
Hemolysin	[108]
Honokiol	[109]
Indoxyl sulfate	[110]
IPA3	[111]
Ipratropium bromide	[112]
Lead	[113]
Leukotriene C(4)	[16]
Lipopeptides	[114]
Listeriolysin	[115]
Lithium	[116]
Lumefantrine	[117]
Lysophosphatidic acid	[9]
Mercury	[118]
Methyldopa	[119]
Methylglyoxal	[120]
Miltefosine	[121]
Mitotane	[122]
Mitoxantrone	[123]
Monensin	[124]
Nitazoxanide	[125]
Novobiocin	[126]
Nystatin	[127]
Ochratoxin A	[128]
Oridonin	[129]
Oxysterol	[130]
Paclitaxel	[131, 132]
PAF	[133]
Parthenolide	[65, 66]
Patulin	[134]
Penta-O-galloyl-*β*-D-glucose	[135]
Peptidoglycan	[136, 137]
Phloretin	[138]
Phorbol-12 myristate-13 acetate	[9]
Phosphate	[139]
Phytic acid	[140]
Plumbagin	[128]
Polyphyllin D	[141]
Probucol	[142]
Prostaglandin E_2_	[29]
Pyrvinium pamoate	[143, 144]
Radiocontrast agents	[145]
Retinoic acid	[146]
Ribavirin	[147]
Rifampicin	[148]
Rotenone	[149]
Salinomycin	[150]
Selenium (sodium selenite)	[151]
Shikonin	[126]
Silver ions	[152]
Sorafenib	[153]
Sphingomyelinase	[154]
Sphingosine	[155]
Sulindac sulfide	[156]
Sunitinib	[157]
Tannic acid	[94]
Tanshinone IIA	[158]
Thioridazine	[159]
Thrombospondin-1-receptor CD47	[160]
Thymoquinone	[161]
Tin	[162]
Trans-cinnamaldehyde	[163]
Tyrosinase	[164]
Ursolic acid	[165]
Valinomycin	[166]
Sodium vanadate	[167]
Vitamin K(3)	[168]
Withaferin A	[169]
Zearalenone	[170]
Zinc	[171]

**Table 2:** Inhibitors of eryptosis.

Inhibitors	References
Adenosine	[172]
Amitriptyline	[173]
Caffeine	[174]
Catecholamines (isoproterenol)	[175]
Chloride	[176]
D4476	[143]
Dibutyryl-cGMP	[17]
Dithiothreitol	[5]
EIPA	[177]
EPO	[19]
Erythropoietin	[19, 178]
Flufenamic acid	[179]
Furosemide	[180]
Glutathione	[38]
7-monohydroxyethylrutoside	[40]
N-acetylcysteine	[18, 20]
Naringin	[181]
NBQX/CNQX	[182]
Niflumic acid	[44]
Nitroprusside (NO-donor)	[17]
NPPB	[44]
Papanonoate (NO-donor)	[17]
P38 Inh III	[183]
Resveratrol	[184]
(R)-DRF053	[143]
Salidroside	[39]
SB203580	[183]
Staurosporine	[14]
Trolox	[31]
Urea	[185]
Vitamin E	[35–37]
Xanthohumol	[186]
Zidovudine	[187]

**Table 3:** Diseases associated with enhanced eryptosis.

Diseases associated with accelerated eryptosis	References
Dehydration	[188]
Hypoxia	[189]
Iron deficiency	[190]
Metabolic syndrome	[191]
Diabetes mellitus	[192]
Phosphate depletion	[193]
Neocytolysis	[21]
Hemolytic anemia	[194]
Heart failure	[195]
Renal insufficiency	[19, 196, 197]
Hemolytic uremic syndrome	[198]
Sepsis	[199]
Mycoplasma infection	[200]
Malaria	[8, 85, 105, 201, 202]
Sickle cell disease	[189, 203–209]
Thalassemia	[203, 204, 206, 210, 211]
Glucose-6-phosphate dehydrogenase (G6PD) deficiency	[203, 212, 213]
Wilson's disease	[81]
AE1 mutation	[214]
GLUT1 mutation	[215]

**Table 4:** Altered eryptosis in gene-targeted mice.

Targeted gene	References
*Enhanced eryptosis *	
GCLM-deficiency	[31]
Annexin 7 deficiency	[216]
Defective hemoglobin (sickle cell, thalassemia)	[205, 206]
cGMP-dependent protein kinase type I (cGKI) deficiency	[217]
AMP-activated protein kinase deficiency	[27]
Klotho deficiency	[218, 219]
EPO excess	[220]
AE1 deficiency	[221]
*Reduced eryptosis *	
PAF receptor deficiency	[133]
Jak3 deficiency	[222]
PDK1 deficiency	[223]
TRPC6 deficiency	[26]

## Abbreviations

AE1: Band 3 or anion exchanger 1
AMPK: AMP-activated kinase (AMPK)
[Ca^2+^]_i_: Cytosolic Ca^2+^ concentration
CD36: Cluster of differentiation 36
cGK: cGMP-dependent protein kinase
CK1*α*: Casein kinase 1*α*
CXCL16/SR-PSOX: CXC-Motiv-Chemokin-16/Scavenger receptor for phosphatidylserine and oxidized low density lipoprotein (SR-PSOX)
GSH: Reduced glutathione
GCLM: Glutamate cysteine ligase modulator
G6PDH: Glucose-6-phosphate dehydrogenase
Hb: Hemoglobin
HUS: Hemolytic uremic syndrome
JAK3: Janus-activated kinase JAK3
L-NAME: L-NG-Nitroarginine Methyl Ester
NADPH: Nicotinamide adenine dinucleotide phosphate
NO: Nitric oxide
PAK2: p21-activated kinase PAK2
PGE_2_: Prostaglandin E_2_
ROS: Reactive oxygen species
SOD: Cu, Zn-superoxide dismutase
TRPC6: Transient receptor potential channel 6
TSP: Thrombospondin-1
VLA-4: Very late-activating antigen-4.

## References

[1] Arese P., Turrini F., Schwarzer E. (2005). Band 3/complement-mediated recognition and removal of normally senescent and pathological human erythrocytes. *Cellular Physiology and Biochemistry*.

[2] Bosman G. J. C. G. M., Willekens F. L. A., Werre J. M. (2005). Erythrocyte aging: a more than superficial resemblance to apoptosis?. *Cellular Physiology and Biochemistry*.

[3] Kiefer C. R., Snyder L. M. (2000). Oxidation and erythrocyte senescence. *Current Opinion in Hematology*.

[4] Lutz H. U. (2004). Innate immune and non-immune mediators of erythrocyte clearance. *Cellular and Molecular Biology*.

[5] Lang F., Lang K. S., Lang P. A., Huber S. M., Wieder T. (2006). Mechanisms and significance of eryptosis. *Antioxidants & Redox Signaling*.

[6] Lang E., Qadri S. M., Lang F. (2012). Killing me softly—suicidal erythrocyte death. *International Journal of Biochemistry and Cell Biology*.

[7] Çimen M. Y. B. (2008). Free radical metabolism in human erythrocytes. *Clinica Chimica Acta*.

[8] Föller M., Bobbala D., Koka S., Huber S. M., Gulbins E., Lang F. (2009). Suicide for survival—death of infected erythrocytes as a host mechanism to survive malaria. *Cellular Physiology and Biochemistry*.

[9] Nguyen D. B., Wagner-Britz L., Maia S. (2011). Regulation of phosphatidylserine exposure in red blood cells. *Cellular Physiology and Biochemistry*.

[10] Lang F., Qadri S. M. (2012). Mechanisms and significance of eryptosis, the suicidal death of erythrocytes. *Blood Purification*.

[11] Lang F., Abed M., Lang E., Föller M. (2014). Oxidative stress and suicidal erythrocyte death. *Antioxidants & Redox Signaling*.

[12] Lang F., Lang E., Fller M. (2012). Physiology and pathophysiology of eryptosis. *Transfusion Medicine and Hemotherapy*.

[13] Lang K. S., Duranton C., Poehlmann H. (2003). Cation channels trigger apoptotic death of erythrocytes. *Cell Death and Differentiation*.

[14] Klarl B. A., Lang P. A., Kempe D. S. (2006). Protein kinase C mediates erythrocyte “programmed cell death” following glucose depletion. *The American Journal of Physiology—Cell Physiology*.

[15] Kamp D., Sieberg T., Haest C. W. M. (2001). Inhibition and stimulation of phospholipid scrambling activity. Consequences for lipid asymmetry, echinocytosis, and microvesiculation of erythrocytes. *Biochemistry*.

[16] Foller M., Mahmud H., Gu S. (2009). Participation of leukotriene C_4_ in the regulation of suicidal erythrocyte death. *Journal of Physiology and Pharmacology*.

[17] Nicolay J. P., Liebig G., Niemoeller O. M. (2008). Inhibition of suicidal erythrocyte death by nitric oxide. *Pflugers Archiv European Journal of Physiology*.

[18] Vota D. M., Crisp R. L., Nesse A. B., Vittori D. C. (2012). Oxidative stress due to aluminum exposure induces eryptosis which is prevented by erythropoietin. *Journal of Cellular Biochemistry*.

[19] Myssina S., Huber S. M., Birka C. (2003). Inhibition of erythrocyte cation channels by erythropoietin. *Journal of the American Society of Nephrology*.

[20] Ghashghaeinia M., Cluitmans J. C. A., Akel A. (2012). The impact of erythrocyte age on eryptosis. *British Journal of Haematology*.

[21] Rice L., Alfrey C. P. (2005). The negative regulation of red cell mass by neocytolysis: physiologic and pathophysiologic manifestations. *Cellular Physiology and Biochemistry*.

[22] Hermle T., Shumilina E., Attanasio P. (2006). Decreased cation channel activity and blunted channel-dependent eryptosis in neonatal erythrocytes. *The American Journal of Physiology—Cell Physiology*.

[23] Jain S. K. (1986). Presence of phosphatidylserine in the outer membrane bilayer of newborn human erythrocytes. *Biochemical and Biophysical Research Communications*.

[24] Setty B. N. Y., Kulkarni S., Rao A. K., Stuart M. J. (2000). Fetal hemoglobin in sickle cell disease: relationship to erythrocyte phosphatidylserine exposure and coagulation activation. *Blood*.

[25] Bell S. G. (1999). An introduction to hemoglobin physiology. *Neonatal Network*.

[26] Föller M., Kasinathan R. S., Koka S. (2008). TRPC6 contributes to the Ca^2+^ leak of human erythrocytes. *Cellular Physiology and Biochemistry*.

[27] Föller M., Sopjani M., Koka S. (2009). Regulation of erythrocyte survival by AMP-activated protein kinase. *The FASEB Journal*.

[28] Duranton C., Huber S. M., Lang F. (2002). Oxidation induces a Cl^−^-dependent cation conductance in human red blood cells. *Journal of Physiology*.

[29] Lang P. A., Kempe D. S., Myssina S. (2005). PGE_2_ in the regulation of programmed erythrocyte death. *Cell Death and Differentiation*.

[30] Kaestner L., Bernhardt I. (2002). Ion channels in the human red blood cell membrane: their further investigation and physiological relevance. *Bioelectrochemistry*.

[31] Föller M., Harris I. S., Elia A. (2013). Functional significance of glutamate-cysteine ligase modifier for erythrocyte survival in vitro and in vivo. *Cell Death and Differentiation*.

[32] Bilmen S., Aksu T. A., Gümüşlü S., Korgun D. K., Canatan D. (2001). Antioxidant capacity of G-6-PD-deficient erythrocytes. *Clinica Chimica Acta*.

[33] Mavelli I., Ciriolo M. R., Rossi L. (1984). Favism: a hemolytic disease associated with increased superoxide dismutase and decreased glutathione peroxidase activities in red blood cells. *European Journal of Biochemistry*.

[34] Damonte G., Guida L., Sdraffa A. (1992). Mechanisms of perturbation of erythrocyte calcium homeostasis in favism. *Cell Calcium*.

[35] Gökkuşu C., Mostafazadeh T. (2003). Changes of oxidative stress in various tissues by long-term administration of vitamin E in hypercholesterolemic rats. *Clinica Chimica Acta*.

[36] Kobayashi S., Moriya H., Aso K., Ohtake T. (2003). Vitamin E-bonded hemodialyzer improves atherosclerosis associated with a rheological improvement of circulating red blood cells. *Kidney International*.

[37] Jain S. K. (1983). Vitamin E and stabilization of membrane lipid organization in red blood cells with peroxidative damage. *Biomedica Biochimica Acta*.

[38] Dumaswala U. J., Wilson M. J., Wu Y. L. (2000). Glutathione loading prevents free radical injury in red blood cells after storage. *Free Radical Research*.

[39] Qian E. W., Ge D. T., Kong S.-K. (2012). Salidroside protects human erythrocytes against hydrogen peroxide-induced apoptosis. *Journal of Natural Products*.

[40] de Franceschi L., Turrini F., Honczarenko M. (2004). In vivo reduction of erythrocyte oxidant stress in a murine model of *β*-thalassemia. *Haematologica*.

[41] Bookchin R. M., Ortiz O. E., Lew V. L. (1987). Activation of calcium-dependent potassium channels in deoxygenated sickled red cells. *Progress in Clinical and Biological Research*.

[42] Brugnara C., De Franceschi L., Alper S. L. (1993). Inhibition of Ca^2+^-dependent K^+^ transport and cell dehydration in sickle erythrocytes by clotrimazole and other imidazole derivatives. *Journal of Clinical Investigation*.

[43] Lang P. A., Kaiser S., Myssina S., Wieder T., Lang F., Huber S. M. (2003). Role of Ca^2+^-activated K^+^ channels in human erythrocyte apoptosis. *The American Journal of Physiology—Cell Physiology*.

[44] Myssina S., Lang P. A., Kempe D. S. (2004). Cl -channel blockers NPPB and niflumic acid blunt Ca^2+^-induced erythrocyte ‘apoptosis’. *Cellular Physiology and Biochemistry*.

[45] Huber S. M., Uhlemann A.-C., Gamper N. L., Duranton C., Kremsner P. G., Lang F. (2002). *Plasmodium falciparum* activates endogenous Cl^−^ channels of human erythrocytes by membrane oxidation. *The EMBO Journal*.

[46] Tanneur V., Duranton C., Brand V. B. (2006). Purinoceptors are involved in the induction of an osmolyte permeability in malaria-infected and oxidized human erythrocytes. *FASEB Journal*.

[47] Lang F., Busch G. L., Ritter M. (1998). Functional significance of cell volume regulatory mechanisms. *Physiological Reviews*.

[48] Berg C. P., Engels I. H., Rothbart A. (2001). Human mature red blood cells express caspase-3 and caspase-8, but are devoid of mitochondrial regulators of apoptosis. *Cell Death and Differentiation*.

[49] Ahmed M. S. E., Langer H., Abed M., Voelkl J., Lang F. (2013). The uremic toxin acrolein promotes suicidal erythrocyte death. *Kidney and Blood Pressure Research*.

[50] Bhavsar S. K., Bobbala D., Xuan N. T., Föller M., Lang F. (2010). Stimulation of suicidal erythrocyte death by *α*-lipoic acid. *Cellular Physiology and Biochemistry*.

[51] Niemoeller O. M., Kiedaisch V., Dreischer P., Wieder T., Lang F. (2006). Stimulation of eryptosis by aluminium ions. *Toxicology and Applied Pharmacology*.

[52] Föller M., Geiger C., Mahmud H., Nicolay J., Lang F. (2008). Stimulation of suicidal erythrocyte death by amantadine. *European Journal of Pharmacology*.

[53] Nicolay J. P., Bentzen P. J., Ghashghaeinia M., Wieder T., Lang F. (2007). Stimulation of erythrocyte cell membrane scrambling by amiodarone. *Cellular Physiology and Biochemistry*.

[54] Mahmud H., Mauro D., Qadri S. M., Föller M., Lang F. (2009). Triggering of suicidal erythrocyte death by Amphotericin B. *Cellular Physiology and Biochemistry*.

[55] Nicolay J. P., Gatz S., Liebig G., Gulbins E., Lang F. (2007). Amyloid induced suicidal erythrocyte death. *Cellular Physiology and Biochemistry*.

[56] Bentzen P. J., Lang F. (2007). Effect of anandamide on erythrocyte survival. *Cellular Physiology and Biochemistry*.

[57] Attanasio P., Shumilina E., Hermle T. (2007). Stimulation of eryptosis by anti-A IgG antibodies. *Cellular Physiology and Biochemistry*.

[58] Zbidah M., Lupescu A., Jilani K. (2012). Apigenin-induced suicidal erythrocyte death. *Journal of Agricultural and Food Chemistry*.

[59] Malik A., Bissinger R., Calabrò S., Faggio C., Jilani K., Lang F. (2014). Aristolochic acid induced suicidal erythrocyte death. *Kidney and Blood Pressure Research*.

[60] Biswas D., Banerjee M., Sen G. (2008). Mechanism of erythrocyte death in human population exposed to arsenic through drinking water. *Toxicology and Applied Pharmacology*.

[61] Mahmud H., Föller M., Lang F. (2009). Arsenic-induced suicidal erythrocyte death. *Archives of Toxicology*.

[62] Alzoubi K., Calabrò S., Bissinger R., Abed M., Faggio C., Lang F. (2014). Stimulation of suicidal erythrocyte death by artesunate. *Cellular Physiology and Biochemistry*.

[63] Bobbala D., Koka S., Geiger C., Föller M., Huber S. M., Lang F. (2009). Azathioprine favourably influences the course of malaria. *Malaria Journal*.

[64] Geiger C., Föller M., Herrlinger K. R., Lang F. (2008). Azathioprine-induced suicidal erythrocyte death. *Inflammatory Bowel Diseases*.

[65] Ghashghaeinia M., Cluitmans J. C., Toulany M. (2013). Age sensitivity of NF*κ*B abundance and programmed cell death in erythrocytes induced by NF*κ*B inhibitors. *Cellular Physiology and Biochemistry*.

[66] Ghashghaeinia M., Toulany M., Saki M. (2011). The NF*κ*B pathway inhibitors bay 11-7082 and parthenolide induce programmed cell death in anucleated erythrocytes. *Cellular Physiology and Biochemistry*.

[67] Shumilina E., Kiedaisch V., Akkel A. (2006). Stimulation of suicidal erythrocyte death by lipoxygenase inhibitor Bay-Y5884. *Cellular Physiology and Biochemistry*.

[68] Qadri S. M., Kucherenko Y., Lang F. (2011). Beauvericin induced erythrocyte cell membrane scrambling. *Toxicology*.

[69] Lang E., Jilani K., Zelenak C. (2011). Stimulation of suicidal erythrocyte death by benzethonium. *Cellular Physiology and Biochemistry*.

[70] Gao M., Lau P. M., Kong S. K. (2014). Mitochondrial toxin betulinic acid induces in vitro eryptosis in human red blood cells through membrane permeabilization. *Archives of Toxicology*.

[71] Braun M., Föller M., Gulbins E., Lang F. (2009). Eryptosis triggered by bismuth. *BioMetals*.

[72] Sopjani M., Föller M., Dreischer P., Lang F. (2008). Stimulation of eryptosis by cadmium ions. *Cellular Physiology and Biochemistry*.

[73] Lang E., Qadri S. M., Jilani K. (2012). Carbon monoxide-sensitive apoptotic death of erythrocytes. *Basic and Clinical Pharmacology and Toxicology*.

[74] Jilani K., Lang F. (2013). Carmustine-induced phosphatidylserine translocation in the erythrocyte membrane. *Toxins*.

[75] Lupescu A., Bissinger R., Jilani K., Lang F. (2013). Triggering of suicidal erythrocyte death by celecoxib. *Toxins*.

[76] Lang K. S., Myssina S., Brand V. (2004). Involvement of ceramide in hyperosmotic shock-induced death of erythrocytes. *Cell Death and Differentiation*.

[77] Akel A., Hermle T., Niemoeller O. M. (2006). Stimulation of erythrocyte phosphatidylserine exposure by chlorpromazine. *European Journal of Pharmacology*.

[78] Lupescu A., Jilani K., Zelenak C., Zbidah M., Qadri S. M., Lang F. (2012). Hexavalent chromium-induced erythrocyte membrane phospholipid asymmetry. *BioMetals*.

[79] Niemoeller O. M., Mahmud H., Föller M., Wieder T., Lang F. (2008). Ciglitazone and 15d-PGJ2 induced suicidal erythrocyte death. *Cellular Physiology and Biochemistry*.

[80] Mahmud H., Föller M., Lang F. (2008). Suicidal erythrocyte death triggered by cisplatin. *Toxicology*.

[81] Lang P. A., Schenck M., Nicolay J. P. (2007). Liver cell death and anemia in Wilson disease involve acid sphingomyelinase and ceramide. *Nature Medicine*.

[82] Lui J. C. K., Wong J. W. Y., Suen Y. K., Kwok T. T., Fung K. P., Kong S. K. (2007). Cordycepin induced eryptosis in mouse erythrocytes through a Ca^2+^-dependent pathway without caspase-3 activation. *Archives of Toxicology*.

[83] Bissinger R., Lupescu A., Zelenak C., Jilani K., Lang F. (2014). Stimulation of eryptosis by cryptotanshinone. *Cellular Physiology and Biochemistry*.

[84] Bentzen P. J., Lang E., Lang F. (2007). Curcumin induced suicidal erythrocyte death. *Cellular Physiology and Biochemistry*.

[85] Bobbala D., Koka S., Lang C., Boini K. M., Huber S. M., Lang F. (2008). Effect of cyclosporine on parasitemia and survival of *Plasmodium berghei* infected mice. *Biochemical and Biophysical Research Communications*.

[86] Niemoeller O. M., Akel A., Lang P. A. (2006). Induction of eryptosis by cyclosporine. *Naunyn-Schmiedeberg's Archives of Pharmacology*.

[87] Mandal D., Mazumder A., Das P., Kundu M., Basu J. (2005). Fas-, caspase 8-, and caspase 3-dependent signaling regulates the activity of the aminophospholipid translocase and phosphatidylserine externalization in human erythrocytes. *Journal of Biological Chemistry*.

[88] Abed M., Zoubi K. A., Theurer M., Lang F. (2013). Effect of dermaseptin on erythrocytes. *Basic and Clinical Pharmacology and Toxicology*.

[89] Qadri S. M., Kucherenko Y., Zelenak C., Jilani K., Lang E., Lang F. (2011). Dicoumarol activates Ca^2+^-permeable cation channels triggering erythrocyte cell membrane scrambling. *Cellular Physiology and Biochemistry*.

[90] Ghashghaeinia M., Bobbala D., Wieder T. (2010). Targeting glutathione by dimethylfumarate protects against experimental malaria by enhancing erythrocyte cell membrane scrambling. *The American Journal of Physiology—Cell Physiology*.

[91] Jilani K., Qadri S. M., Lang E. (2011). Stimulation of erythrocyte phospholipid scrambling by enniatin A. *Molecular Nutrition and Food Research*.

[92] Bissinger R., Modicano P., Frauenfeld L. (2013). Estramustine-induced suicidal erythrocyte death. *Cellular Physiology and Biochemistry*.

[93] Gao M., Wong S. Y., Lau P. M., Kong S. K. (2013). Ferutinin induces in vitro eryptosis/erythroptosis in human erythrocytes through membrane permeabilization and calcium influx. *Chemical Research in Toxicology*.

[94] Jilani K., Enkel S., Bissinger R., Almilaji A., Abed M., Lang F. (2013). Fluoxetine induced suicidal erythrocyte death. *Toxins*.

[95] Eberhard M., Ferlinz K., Alizzi K. (2010). FTY720-induced suicidal erythrocyte death. *Cellular Physiology and Biochemistry*.

[96] Zbidah M., Lupescu A., Jilani K., Lang F. (2013). Stimulation of suicidal erythrocyte death by fumagillin. *Basic and Clinical Pharmacology and Toxicology*.

[97] Lupescu A., Jilani K., Zelenak C., Zbidah M., Shaik N., Lang F. (2012). Induction of programmed erythrocyte death by gambogic acid. *Cellular Physiology and Biochemistry*.

[98] Lupescu A., Bissinger R., Warsi J., Jilani K., Lang F. (2014). Stimulation of erythrocyte cell membrane scrambling by gedunin. *Cellular Physiology and Biochemistry*.

[99] Jilani K., Qadri S. M., Lang F. (2013). Geldanamycin-induced phosphatidylserine translocation in the erythrocyte membrane. *Cellular Physiology and Biochemistry*.

[100] Kucherenko Y. V., Bhavsar S. K., Grischenko V. I., Fischer U. R., Huber S. M., Lang F. (2010). Increased cation conductance in human erythrocytes artificially aged by glycation. *Journal of Membrane Biology*.

[101] Head D. J., Lee Z. E., Poole J., Avent N. D. (2005). Expression of phosphatidylserine (PS) on wild-type and Gerbich variant erythrocytes following glycophorin-C (GPC) ligation. *British Journal of Haematology*.

[102] Sopjani M., Föller M., Lang F. (2008). Gold stimulates Ca^2+^ entry into and subsequent suicidal death of erythrocytes. *Toxicology*.

[103] Lau I. P., Chen H., Wang J. (2012). In vitro effect of CTAB-and PEG-coated gold nanorods on the induction of eryptosis/erythroptosis in human erythrocytes. *Nanotoxicology*.

[104] Zbidah M., Lupescu A., Shaik N., Lang F. (2012). Gossypol-induced suicidal erythrocyte death. *Toxicology*.

[105] Böttger E., Multhoff G., Kun J. F. J., Esen M. (2012). *Plasmodium falciparum*-infected erythrocytes induce granzyme B by NK cells through expression of host-Hsp70. *PLoS ONE*.

[106] Gatidis S., Föller M., Lang F. (2009). Hemin-induced suicidal erythrocyte death. *Annals of Hematology*.

[107] Zhang R., Xiang Y., Ran Q. (2014). Involvement of calcium, reactive oxygen species, and ATP in hexavalent chromium-induced damage in red blood cells. *Cellular Physiology and Biochemistry*.

[108] Lang P. A., Kaiser S., Myssina S. (2004). Effect of Vibrio parahaemolyticus haemolysin on human erythrocytes. *Cellular Microbiology*.

[109] Zbidah M., Lupescu A., Herrmann T. (2013). Effect of honokiol on erythrocytes. *Toxicology in Vitro*.

[110] Ahmed M. S. E., Abed M., Voelkl J., Lang F. (2013). Triggering of suicidal erythrocyte death by uremic toxin indoxyl sulfate. *BMC Nephrology*.

[111] Zelenak C., Föller M., Velic A. (2011). Proteome analysis of erythrocytes lacking AMP-activated protein kinase reveals a role of PAK2 kinase in eryptosis. *Journal of Proteome Research*.

[112] Shaik N., Alhourani E., Bosc A. (2012). Stimulation of suicidal erythrocyte death by ipratropium bromide. *Cellular Physiology and Biochemistry*.

[113] Kempe D. S., Lang P. A., Eisele K. (2005). Stimulation of erythrocyte phosphatidylserine exposure by lead ions. *The American Journal of Physiology—Cell Physiology*.

[114] Wang K., Mahmud H., Föller M. (2008). Lipopeptides in the triggering of erythrocyte cell membrane scrambling. *Cellular Physiology and Biochemistry*.

[115] Föller M., Shumilina E., Lam R. S. (2007). Induction of suicidal erythrocyte death by listeriolysin from *Listeria monocytogenes*. *Cellular Physiology and Biochemistry*.

[116] Nicolay J. P., Gatz S., Lang F., Lang U. E. (2010). Lithium-induced suicidal erythrocyte death. *Journal of Psychopharmacology*.

[117] Alzoubi K., Alktifan B., Oswald G., Fezai M., Abed M., Lang F. (2014). Breakdown of phosphatidylserine asymmetry following treatment of erythrocytes with lumefantrine. *Toxins*.

[118] Eisele K., Lang P. A., Kempe D. S. (2006). Stimulation of erythrocyte phosphatidylserine exposure by mercury ions. *Toxicology and Applied Pharmacology*.

[119] Mahmud H., Föller M., Lang F. (2008). Stimulation of erythrocyte cell membrane scrambling by methyldopa. *Kidney and Blood Pressure Research*.

[120] Nicolay J. P., Schneider J., Niemoeller O. M. (2006). Stimulation of suicidal erythrocyte death by methylglyoxal. *Cellular Physiology and Biochemistry*.

[121] Munoz C., Alzoubi K., Jacobi J., Abed M., Lang F. (2013). Effect of miltefosine on erythrocytes. *Toxicology in Vitro*.

[122] Jacobi J., Lang E., Bissinger R. (2014). Stimulation of erythrocyte cell membrane scrambling by mitotane. *Cellular Physiology and Biochemistry*.

[123] Arnold M., Bissinger R., Lang F. (2014). Mitoxantrone-induced suicidal erythrocyte death. *Cellular Physiology and Biochemistry*.

[124] Bhavsar S. K., Eberhard M., Bobbala D., Lang F. (2010). Monensin induced suicidal erythrocyte death. *Cellular Physiology and Biochemistry*.

[125] Arnold M., Lang E., Modicano P. (2014). Effect of nitazoxanide on erythrocytes. *Basic and Clinical Pharmacology and Toxicology*.

[126] Lupescu A., Bissinger R., Herrmann T., Oswald G., Jilani K., Lang F. (2014). Induction of suicidal erythrocyte death by novobiocin. *Cellular Physiology and Biochemistry*.

[127] Malik A., Bissinger R., Jilani K., Lang F. (2015). Stimulation of erythrocyte cell membrane scrambling by nystatin. *Basic & Clinical Pharmacology & Toxicology*.

[128] Jilani K., Lupescu A., Zbidah M., Abed M., Shaik N., Lang F. (2012). Enhanced apoptotic death of erythrocytes induced by the mycotoxin ochratoxin A. *Kidney and Blood Pressure Research*.

[129] Jilani K., Qadri S. M., Zelenak C., Lang F. (2011). Stimulation of suicidal erythrocyte death by oridonin. *Archives of Biochemistry and Biophysics*.

[130] Tesoriere L., Attanzio A., Allegra M., Cilla A., Gentile C., Livrea M. A. (2014). Oxysterol mixture in hypercholesterolemia-relevant proportion causes oxidative stress-dependent eryptosis. *Cellular Physiology and Biochemistry*.

[131] Koka S., Bobbala D., Lang C., Boini K. M., Huber S. M., Lang F. (2009). Influence of paclitaxel on parasitemia and survival of *Plasmodium berghei* infected mice. *Cellular Physiology and Biochemistry*.

[132] Lang P. A., Huober J., Bachmann C. (2006). Stimulation of erythrocyte phosphatidylserine exposure by paclitaxel. *Cellular Physiology and Biochemistry*.

[133] Lang P. A., Kempe D. S., Tanneur V. (2005). Stimulation of erythrocyte ceramide formation by platelet-activating factor. *Journal of Cell Science*.

[134] Lupescu A., Jilani K., Zbidah M., Lang F. (2013). Patulin-induced suicidal erythrocyte death. *Cellular Physiology and Biochemistry*.

[135] Alzoubi K., Honisch S., Abed M., Lang F. (2014). Triggering of suicidal erythrocyte death by penta-*O*-galloyl-beta-D-glucose. *Toxins*.

[136] Abed M., Towhid S. T., Pakladok T. (2013). Effect of bacterial peptidoglycan on erythrocyte death and adhesion to endothelial cells. *International Journal of Medical Microbiology*.

[137] Föller M., Biswas R., Mahmud H. (2009). Effect of peptidoglycans on erythrocyte survival. *International Journal of Medical Microbiology*.

[138] Bissinger R., Fischer S., Jilani K., Lang F. (2014). Stimulation of erythrocyte death by phloretin. *Cellular Physiology and Biochemistry*.

[139] Voelkl J., Alzoubi K., Mamar A.-K., Ahmed M. S. E., Abed M., Lang F. (2014). Stimulation of suicidal erythrocyte death by increased extracellular phosphate concentrations. *Kidney and Blood Pressure Research*.

[140] Eberhard M., Föller M., Lang F. (2010). Effect of phytic acid on suicidal erythrocyte death. *Journal of Agricultural and Food Chemistry*.

[141] Gao M., Cheung K. L., Lau I. P. (2012). Polyphyllin D induces apoptosis in human erythrocytes through Ca^2+^ rise and membrane permeabilization. *Archives of Toxicology*.

[142] Shaik N., Lupescu A., Lang F. (2013). Inhibition of suicidal erythrocyte death by probucol. *Journal of Cardiovascular Pharmacology*.

[143] Zelenak C., Eberhard M., Jilani K., Qadri S. M., MacEk B., Lang F. (2012). Protein kinase CK1*α* regulates erythrocyte survival. *Cellular Physiology and Biochemistry*.

[144] Kucherenko Y., Zelenak C., Eberhard M., Qadri S. M., Lang F. (2012). Effect of casein kinase 1alpha activator pyrvinium pamoate on erythrocyte ion channels. *Cellular Physiology and Biochemistry*.

[145] Föller M., Sopjani M., Schlemmer H. P., Claussen C. D., Lang F. (2009). Triggering of suicidal erythrocyte death by radiocontrast agents. *European Journal of Clinical Investigation*.

[146] Niemoeller O. M., Föller M., Lang C., Huber S. M., Lang F. (2008). Retinoic acid induced suicidal erythrocyte death. *Cellular Physiology and Biochemistry*.

[147] Oswald G., Alzoubi K., Abed M., Lang F. (2014). Stimulation of suicidal erythrocyte death by ribavirin. *Basic & Clinical Pharmacology & Toxicology*.

[148] Abed M., Towhid S. T., Shaik N., Lang F. (2012). Stimulation of suicidal death of erythrocytes by rifampicin. *Toxicology*.

[149] Lupescu A., Jilani K., Zbidah M., Lang F. (2012). Induction of apoptotic erythrocyte death by rotenone. *Toxicology*.

[150] Bissinger R., Malik A., Jilani K., Lang F. (2014). Triggering of erythrocyte cell membrane scrambling by salinomycin. *Basic & Clinical Pharmacology & Toxicology*.

[151] Sopjani M., Föller M., Gulbins E., Lang F. (2008). Suicidal death of erythrocytes due to selenium-compounds. *Cellular Physiology and Biochemistry*.

[152] Sopjani M., Föller M., Haendeler J., Götz F., Lang F. (2009). Silver ion-induced suicidal erythrocyte death. *Journal of Applied Toxicology*.

[153] Lupescu A., Shaik N., Jilani K. (2012). Enhanced erythrocyte membrane exposure of phosphatidylserine following sorafenib treatment: an *in vivo* and *in vitro* study. *Cellular Physiology and Biochemistry*.

[154] Abed M., Towhid S. T., Mia S. (2012). Sphingomyelinase-induced adhesion of eryptotic erythrocytes to endothelial cells. *American Journal of Physiology—Cell Physiology*.

[155] Qadri S. M., Bauer J., Zelenak C. (2011). Sphingosine but not sphingosine-1-phosphate stimulates suicidal erythrocyte death. *Cellular Physiology and Biochemistry*.

[156] Zbidah M., Lupescu A., Yang W. (2012). Sulindac sulfde—induced stimulation of eryptosis. *Cellular Physiology and Biochemistry*.

[157] Shaik N., Lupescu A., Lang F. (2012). Sunitinib-sensitive suicidal erythrocyte death. *Cellular Physiology and Biochemistry*.

[158] Zelenak C., Pasham V., Jilani K. (2012). Tanshinone IIA stimulates erythrocyte phosphatidylserine exposure. *Cellular Physiology and Biochemistry*.

[159] Lang E., Modicano P., Arnold M. (2013). Effect of thioridazine on erythrocytes. *Toxins*.

[160] Head D. J., Lee Z. E., Swallah M. M., Avent N. D. (2005). Ligation of CD47 mediates phosphatidylserine expression on erythrocytes and a concomitant loss of viability in vitro. *British Journal of Haematology*.

[161] Qadri S. M., Mahmud H., Föller M., Lang F. (2009). Thymoquinone-induced suicidal erythrocyte death. *Food and Chemical Toxicology*.

[162] Nguyen T. T., Föller M., Lang F. (2009). Tin triggers suicidal death of erythrocytes. *Journal of Applied Toxicology*.

[163] Theurer M., Shaik N., Lang F. (2013). Stimulation of suicidal erythrocyte death by trans-cinnamaldehyde. *Phytomedicine*.

[164] Frauenfeld L., Alzoubi K., Abed M., Lang F. (2014). Stimulation of erythrocyte cell membrane scrambling by mushroom tyrosinase. *Toxins*.

[165] Jilani K., Abed M., Zelenak C., Lang E., Qadri S. M., Lang F. (2011). Triggering of erythrocyte cell membrane scrambling by ursolic acid. *Journal of Natural Products*.

[166] Schneider J., Nicolay J. P., Föller M., Wieder T., Lang F. (2007). Suicidal erythrocyte death following cellular K^+^ loss. *Cellular Physiology and Biochemistry*.

[167] Föller M., Sopjani M., Mahmud H., Lang F. (2008). Vanadate-induced suicidal erythrocyte death. *Kidney and Blood Pressure Research*.

[168] Qadri S. M., Eberhard M., Mahmud H., Föller M., Lang F. (2009). Stimulation of ceramide formation and suicidal erythrocyte death by vitamin K3 (menadione). *European Journal of Pharmacology*.

[169] Jilani K., Lupescu A., Zbidah M., Shaik N., Lang F. (2013). Withaferin A-stimulated Ca^2+^ entry, ceramide formation and suicidal death of erythrocytes. *Toxicology in Vitro*.

[170] Jilani K., Lang F. (2013). Ca^2+^-dependent suicidal erythrocyte death following zearalenone exposure. *Archives of Toxicology*.

[171] Kiedaisch V., Akel A., Niemoeller O. M., Wieder T., Lang F. (2008). Zinc-induced suicidal erythrocyte death. *American Journal of Clinical Nutrition*.

[172] Niemoeller O. M., Bentzen P. J., Lang E., Lang F. (2007). Adenosine protects against suicidal erythrocyte death. *Pflügers Archiv*.

[173] Brand V., Koka S., Lang C. (2008). Influence of amitriptyline on eryptosis, parasitemia and survival of *Plasmodium berghei*-infected mice. *Cellular Physiology and Biochemistry*.

[174] Floride E., Föller M., Ritter M., Lang F. (2008). Caffeine inhibits suicidal erythrocyte death. *Cellular Physiology and Biochemistry*.

[175] Lang P. A., Kempe D. S., Akel A. (2005). Inhibition of erythrocyte 'apoptosis' by catecholamines. *Naunyn-Schmiedeberg's Archives of Pharmacology*.

[176] Huber S. M., Gamper N., Lang F. (2001). Chloride conductance and volume-regulatory nonselective cation conductance in human red blood cell ghosts. *Pflugers Archiv*.

[177] Lang K. S., Myssina S., Tanneur V. (2003). Inhibition of erythrocyte cation channels and apoptosis by ethylisopropylamiloride. *Naunyn-Schmiedeberg's Archives of Pharmacology*.

[178] Vota D. M., Maltaneri R. E., Wenker S. D., Nesse A. B., Vittori D. C. (2013). Differential erythropoietin action upon cells induced to eryptosis by different agents. *Cell Biochemistry and Biophysics*.

[179] Kasinathan R. S., Föller M., Koka S., Huber S. M., Lang F. (2007). Inhibition of eryptosis and intraerythrocytic growth of *Plasmodium falciparum* by flufenamic acid. *Naunyn-Schmiedeberg's Archives of Pharmacology*.

[180] Kucherenko Y. V., Lang F. (2012). Inhibitory effect of furosemide on non-selective voltage-independent cation channels in human erythrocytes. *Cellular Physiology and Biochemistry*.

[181] Shaik N., Zbidah M., Lang F. (2012). Inhibition of Ca^2+^ entry and suicidal erythrocyte death by naringin. *Cellular Physiology and Biochemistry*.

[182] Föller M., Mahmud H., Gu S. (2009). Modulation of suicidal erythrocyte cation channels by an AMPA antagonist. *Journal of Cellular and Molecular Medicine*.

[183] Gatidis S., Zelenak C., Fajol A. (2011). P38 MAPK activation and function following osmotic shock of erythrocytes. *Cellular Physiology and Biochemistry*.

[184] Qadri S. M., Föller M., Lang F. (2009). Inhibition of suicidal erythrocyte death by resveratrol. *Life Sciences*.

[185] Lang K. S., Myssina S., Lang P. A. (2004). Inhibition of erythrocyte phosphatidylserine exposure by urea and Cl^−^. *American Journal of Physiology—Renal Physiology*.

[186] Qadri S. M., Mahmud H., Föller M., Lang F. (2009). Inhibition of suicidal erythrocyte death by xanthohumol. *Journal of Agricultural and Food Chemistry*.

[187] Kucherenko Y., Geiger C., Shumilina E., Föller M., Lang F. (2008). Inhibition of cation channels and suicidal death of human erythrocytes by zidovudine. *Toxicology*.

[188] Abed M., Feger M., Alzoubi K. (2013). Sensitization of erythrocytes to suicidal erythrocyte death following water deprivation. *Kidney and Blood Pressure Research*.

[189] Weiss E., Cytlak U. M., Rees D. C., Osei A., Gibson J. S. (2012). Deoxygenation-induced and Ca^2+^ dependent phosphatidylserine externalisation in red blood cells from normal individuals and sickle cell patients. *Cell Calcium*.

[190] Kempe D. S., Lang P. A., Duranton C. (2006). Enhanced programmed cell death of iron-deficient erythrocytes. *The FASEB Journal*.

[191] Zappulla D. (2008). Environmental stress, erythrocyte dysfunctions, inflammation, and the metabolic syndrome: adaptations to CO_2_ increases?. *Journal of the Cardiometabolic Syndrome*.

[192] Fırat U., Kaya S., Çim A. (2012). Increased caspase-3 immunoreactivity of erythrocytes in STZ diabetic rats. *Experimental Diabetes Research*.

[193] Birka C., Lang P. A., Kempe D. S. (2004). Enhanced susceptibility to erythrocyte ‘apoptosis’ following phosphate depletion. *Pflügers Archiv*.

[194] Banerjee D., Saha S., Basu S., Chakrabarti A. (2008). Porous red cell ultrastructure and loss of membrane asymmetry in a novel case of hemolytic anemia. *European Journal of Haematology*.

[195] Mahmud H., Ruifrok W. P. T., Westenbrink B. D. (2013). Suicidal erythrocyte death, eryptosis, as a novel mechanism in heart failure-associated anaemia. *Cardiovascular Research*.

[196] Polak-Jonkisz D., Purzyc L. (2012). Ca^2+^ influx versus efflux during eryptosis in uremic erythrocytes. *Blood Purification*.

[197] Abed M., Artunc F., Alzoubi K. (2014). Suicidal erythrocyte death in end-stage renal disease. *Journal of Molecular Medicine*.

[198] Lang P. A., Beringer O., Nicolay J. P. (2006). Suicidal death of erythrocytes in recurrent hemolytic uremic syndrome. *Journal of Molecular Medicine*.

[199] Kempe D. S., Akel A., Lang P. A. (2007). Suicidal erythrocyte death in sepsis. *Journal of Molecular Medicine*.

[200] Felder K. M., Hoelzle K., Ritzmann M. (2011). Hemotrophic mycoplasmas induce programmed cell death in red blood cells. *Cellular Physiology and Biochemistry*.

[201] Koka S., Lang C., Niemoeller O. M. (2008). Influence of NO synthase inhibitor L-NAME on parasitemia and survival of *Plasmodium berghei* infected mice. *Cellular Physiology and Biochemistry*.

[202] Totino P. R. R., Daniel-Ribeiro C. T., Ferreira-da-Cru M. D. F. (2011). Refractoriness of eryptotic red blood cells to plasmodium falciparum infection: a putative host defense mechanism limiting parasitaemia. *PLoS ONE*.

[203] Lang K. S., Roll B., Myssina S. (2002). Enhanced erythrocyte apoptosis in sickle cell anemia, thalassemia and glucose-6-phosphate dehydrogenase deficiency. *Cellular Physiology and Biochemistry*.

[204] Ayi K., Turrini F., Piga A., Arese P. (2004). Enhanced phagocytosis of ring-parasitized mutant erythrocytes: a common mechanism that may explain protection against falciparum malaria in sickle trait and beta-thalassemia trait. *Blood*.

[205] de Jong K., Emerson R. K., Butler J., Bastacky J., Mohandas N., Kuypers F. A. (2001). Short survival of phosphatidylserine-exposing red blood cells in murine sickle cell anemia. *Blood*.

[206] Kean L. S., Brown L. E., Nichols J. W., Mohandas N., Archer D. R., Hsu L. L. (2002). Comparison of mechanisms of anemia in mice with sickle cell disease and beta-thalassemia: peripheral destruction, ineffective erythropoiesis, and phospholipid scramblase-mediated phosphatidylserine exposure. *Experimental Hematology*.

[207] Browning J. A., Robinson H. C., Ellory J. C., Gibson J. S. (2007). Deoxygenation-induced non-electrolyte pathway in red cells from sickle cell patients. *Cellular Physiology and Biochemistry*.

[208] Wood B. L., Gibson D. F., Tait J. F. (1996). Increased erythrocyte phosphatidylserine exposure in sickle cell disease: flow-cytometric measurement and clinical associations. *Blood*.

[209] Chadebech P., Habibi A., Nzouakou R. (2009). Delayed hemolytic transfusion reaction in sickle cell disease patients: evidence of an emerging syndrome with suicidal red blood cell death. *Transfusion*.

[210] Kuypers F. A., Yuan J., Lewis R. A. (1998). Membrane phospholipid asymmetry in human thalassemia. *Blood*.

[211] Basu S., Banerjee D., Chandra S., Chakrabarti A. (2010). Eryptosis in hereditary spherocytosis and thalassemia: role of glycoconjugates. *Glycoconjugate Journal*.

[212] Cappadoro M., Giribaldi G., O'Brien E. (1998). Early phagocytosis of glucose-6-phosphate dehydrogenase (G6PD)-deficient erythrocytes parasitized by *Plasmodium falciparum* may explain malaria protection in G6PD deficiency. *Blood*.

[213] Ganesan S., Chaurasiya N. D., Sahu R., Walker L. A., Tekwani B. L. (2012). Understanding the mechanisms for metabolism-linked hemolytic toxicity of primaquine against glucose 6-phosphate dehydrogenase deficient human erythrocytes: evaluation of eryptotic pathway. *Toxicology*.

[214] Bruce L. J., Robinson H. C., Guizouarn H. (2005). Monovalent cation leaks in human red cells caused by single amino-acid substitutions in the transport domain of the band 3 chloride-bicarbonate exchanger, AE1. *Nature Genetics*.

[215] Weber Y. G., Storch A., Wuttke T. V. (2008). GLUT1 mutations are a cause of paroxysmal exertion-induced dyskinesias and induce hemolytic anemia by a cation leak. *The Journal of Clinical Investigation*.

[216] Abed M., Balasaheb S., Towhid S. T., Daniel C., Amann K., Lang F. (2013). Adhesion of annexin 7 deficient erythrocytes to endothelial cells. *PLoS ONE*.

[217] Föller M., Feil S., Ghoreschi K. (2008). Anemia and splenomegaly in cGKI-deficient mice. *Proceedings of the National Academy of Sciences of the United States of America*.

[218] Abed M., Towhid S. T., Feger M. (2013). Adhesion of klotho-deficient eryptotic erythrocytes to endothelial cells. *Acta Physiologica*.

[219] Kempe D. S., Ackermann T. F., Fischer S. S. (2009). Accelerated suicidal erythrocyte death in Klotho-deficient mice. *Pflugers Archiv European Journal of Physiology*.

[220] Föller M., Kasinathan R. S., Koka S. (2007). Enhanced susceptibility to suicidal death of erythrocytes from transgenic mice overexpressing erythropoietin. *The American Journal of Physiology—Regulatory Integrative and Comparative Physiology*.

[221] Akel A., Wagner C. A., Kovacikova J. (2007). Enhanced suicidal death of erythrocytes from gene-targeted mice lacking the Cl^−^/HCO_3_
^−^ exchanger AE1. *The American Journal of Physiology—Cell Physiology*.

[222] Bhavsar S. K., Gu S., Bobbala D., Lang F. (2011). Janus kinase 3 is expressed in erythrocytes, phosphorylated upon energy depletion and involved in the regulation of suicidal erythrocyte death. *Cellular Physiology and Biochemistry*.

[223] Föller M., Mahmud H., Koka S., Lang F. (2008). Reduced Ca^2+^ entry and suicidal death of erythrocytes in PDK1 hypomorphic mice. *Pflugers Archiv European Journal of Physiology*.

[224] Maellaro E., Leoncini S., Moretti D. (2013). Erythrocyte caspase-3 activation and oxidative imbalance in erythrocytes and in plasma of type 2 diabetic patients. *Acta Diabetologica*.

[225] Mandal D., Baudin-Creuza V., Bhattacharyya A. (2003). Caspase 3-mediated proteolysis of the N-terminal cytoplasmic domain of the human erythroid anion exchanger 1 (band 3). *Journal of Biological Chemistry*.

[226] Bratosin D., Estaquier J., Petit F. (2001). Programmed cell death in mature erythrocytes: a model for investigating death effector pathways operating in the absence of mitochondria. *Cell Death and Differentiation*.

[227] Mandal D., Moitra P. K., Saha S., Basu J. (2002). Caspase 3 regulates phosphatidylserine externalization and phagocytosis of oxidatively stressed erythrocytes. *FEBS Letters*.

[228] Matarrese P., Straface E., Pietraforte D. (2005). Peroxynitrite induces senescence and apoptosis of red blood cells through the activation of aspartyl and cysteinyl proteases. *The FASEB Journal*.

[229] Weil M., Jacobson M. D., Raff M. C. (1998). Are caspases involved in the death of cells with a transcriptionally inactive nucleus? Sperm and chicken erythrocytes. *Journal of Cell Science*.

[230] Das A., Smolenski A., Lohmann S. M., Kukreja R. C. (2006). Cyclic GMP-dependent protein kinase I*α* attenuates necrosis and apoptosis following ischemia/reoxygenation in adult cardiomyocyte. *Journal of Biological Chemistry*.

[231] Li J. R., Billiar T. R. (1999). The anti-apoptotic actions of nitric oxide in hepatocytes. *Cell Death and Differentiation*.

[232] Nagai-Kusuhara A., Nakamura M., Mukuno H., Kanamori A., Negi A., Seigel G. M. (2007). cAMP-responsive element binding protein mediates a cGMP/protein kinase G-deendent anti-apoptotic signal induced by nitric oxide in retinal neuro-glial progenitor cells. *Experimental Eye Research*.

[233] Schlossmann J., Feil R., Hofmann F. (2005). Insights into cGMP signalling derived from cGMP kinase knockout mice. *Frontiers in Bioscience*.

[234] Owusu B. Y., Stapley R., Patel R. P. (2012). Nitric oxide formation versus scavenging: the red blood cell balancing act. *Journal of Physiology (London)*.

[235] Kahn M. J., Maley J. H., Lasker G. F., Kadowitz P. J. (2013). Updated role of nitric oxide in disorders of erythrocyte function. *Cardiovascular and Hematological Disorders—Drug Targets*.

[236] Gow A. J., Luchsinger B. P., Pawloski J. R., Singel D. J., Stamler J. S. (1999). The oxyhemoglobin reaction of nitric oxide. *Proceedings of the National Academy of Sciences of the United States of America*.

[237] Malan D., Levi R. C., Alloatti G., Marcantoni A., Bedendi I., Gallo M. P. (2003). Cyclic AMP and cyclic GMP independent stimulation of ventricular calcium current by peroxynitrite donors in guinea pig myocytes. *Journal of Cellular Physiology*.

[238] William M., Vien J., Hamilton E. (2005). The nitric oxide donor sodium nitroprusside stimulates the Na^+^-K^+^ pump in isolated rabbit cardiac myocytes. *Journal of Physiology*.

[239] Dimmeler S., Haendeler J., Nehls M., Zeiher A. M. (1997). Suppression of apoptosis by nitric oxide via inhibition of interleukin- 1*β*-converting enzyme (ICE)-like and cysteine protease protein (CPP)-32-like proteases. *The Journal of Experimental Medicine*.

[240] Rössig L., Fichtlscherer B., Breitschopf K. (1999). Nitric oxide inhibits caspase-3 by S-nitrosation in vivo. *Journal of Biological Chemistry*.

[241] Benhar M., Stamler J. S. (2005). A central role for S-nitrosylation in apoptosis. *Nature Cell Biology*.

[242] Haendeler J., Hoffmann J., Tischler V., Berk B. C., Zeiher A. M., Dimmeler S. (2002). Redox regulatory and anti-apoptotic functions of thioredoxin depend on S-nitrosylation at cysteine 69. *Nature Cell Biology*.

[243] Haendeler J., Hoffmann J., Zeiher A. M., Dimmeler S. (2004). Antioxidant effects of statins via *S*-nitrosylation and activation of thioredoxin in endothelial cells: a novel vasculoprotective function of statins. *Circulation*.

[244] Hoffmann J., Haendeler J., Zeiher A. M., Dimmeler S. (2001). TNF alpha and oxLDL reduce protein S-nitrosylation in endothelial cells. *Journal of Biological Chemistry*.

[245] Melino G., Bernassola F., Knight R. A., Corasaniti M. T., Nisticò G., Finazzi-Agrò A. (1997). S-nitrosylation regulates apoptosis. *Nature*.

[246] Castillo S. S., Levy M., Wang C., Thaikoottathil J. V., Khan E., Goldkorn T. (2007). Nitric oxide-enhanced caspase-3 and acidic sphingomyelinase interaction: a novel mechanism by which airway epithelial cells escape ceramide-induced apoptosis. *Experimental Cell Research*.

[247] Davidson S. M., Duchen M. R. (2007). Endothelial mitochondria: contributing to vascular function and disease. *Circulation Research*.

[248] Pacher P., Beckman J. S., Liaudet L. (2007). Nitric oxide and peroxynitrite in health and disease. *Physiological Reviews*.

[249] Dasgupta T., Fabry M. E., Kaul D. K. (2010). Antisickling property of fetal hemoglobin enhances nitric oxide bioavailability and ameliorates organ oxidative stress in transgenic-knockout sickle mice. *The American Journal of Physiology—Regulatory Integrative and Comparative Physiology*.

[250] Salhany J. M. (2013). The oxidative denitrosylation mechanism and nitric oxide release from human fetal and adult hemoglobin, an experimentally based model simulation study. *Blood Cells, Molecules, and Diseases*.

[251] Kaul D. K., Liu X.-D., Chang H.-Y., Nagel R. L., Fabry M. E. (2004). Effect of fetal hemoglobin on microvascular regulation in sickle transgenic-knockout mice. *Journal of Clinical Investigation*.

[252] Borst O., Abed M., Alesutan I. (2012). Dynamic adhesion of eryptotic erythrocytes to endothelial cells via CXCL16/SR-PSOX. *The American Journal of Physiology—Cell Physiology*.

[253] Betal S. G., Setty B. N. Y. (2008). Phosphatidylserine-positive erythrocytes bind to immobilized and soluble thrombospondin-1 via its heparin-binding domain. *Translational Research*.

[254] Manodori A. B., Barabino G. A., Lubin B. H., Kuypers F. A. (2000). Adherence of phosphatidylserine-exposing erythrocytes to endothelial matrix thrombospondin. *Blood*.

[255] Fadok V. A., Bratton D. L., Rose D. M., Pearson A., Ezekewitz R. A. B., Henson P. M. (2000). A receptor for phosphatidylserine-specific clearance of apoptotic cells. *Nature*.

[256] Walker B., Towhid S. T., Schmid E. (2014). Dynamic adhesion of eryptotic erythrocytes to immobilized platelets via platelet phosphatidylserine receptors. *The American Journal of Physiology—Cell Physiology*.

[257] Andrews D. A., Low P. S. (1999). Role of red blood cells in thrombosis. *Current Opinion in Hematology*.

[258] Nagababu E., Gulyani S., Earley C. J., Cutler R., Mattson M., Rifkind J. (2008). Iron-deficiency anaemia enhances red blood cell oxidative stress. *Free Radical Research*.

[259] Qadri S. M., Mahmud H., Lang E. (2012). Enhanced suicidal erythrocyte death in mice carrying a loss-of-function mutation of the *adenomatous polyposis coli* gene. *Journal of Cellular and Molecular Medicine*.

[260] Calderón-Salinas J. V., Muñoz-Reyes E. G., Guerrero-Romero J. F. (2011). Eryptosis and oxidative damage in type 2 diabetic mellitus patients with chronic kidney disease. *Molecular and Cellular Biochemistry*.

[261] Thornalley P. J. (1988). Modification of the glyoxalase system in human red blood cells by glucose in vitro. *Biochemical Journal*.

[262] Nwose E. U., Jelinek H. F., Richards R. S., Kerr R. G. (2007). Erythrocyte oxidative stress in clinical management of diabetes and its cardiovascular complications. *British Journal of Biomedical Science*.

[263] Bandeira S. D. M., Guedes G. D. S., de Fonseca L. J. S. (2012). Characterization of blood oxidative stress in type 2 diabetes mellitus patients: increase in lipid peroxidation and SOD activity. *Oxidative Medicine and Cellular Longevity*.

[264] Dmitrieva O., de Lusignan S., Macdougall I. C. (2013). Association of anaemia in primary care patients with chronic kidney disease: cross sectional study of quality improvement in chronic kidney disease (QICKD) trial data. *BMC Nephrology*.

[265] Yang M., Fox C. H., Vassalotti J., Choi M. (2011). Complications of progression of CKD. *Advances in Chronic Kidney Disease*.

[266] Adamson J. W. (2009). Hyporesponsiveness to erythropoiesis stimulating agents in chronic kidney disease: the many faces of inflammation. *Advances in Chronic Kidney Disease*.

[267] Atkinson M. A., Furth S. L. (2011). Anemia in children with chronic kidney disease. *Nature Reviews Nephrology*.

[268] Attanasio P., Ronco C., Anker S. D., Cicoira M., von Haehling S. (2012). Role of iron deficiency and anemia in cardio-renal syndromes. *Seminars in Nephrology*.

[269] Besarab A., Coyne D. W. (2010). Iron supplementation to treat anemia in patients with chronic kidney disease. *Nature Reviews Nephrology*.

[270] Kovesdy C. P. (2009). Iron and clinical outcomes in dialysis and non-dialysis-dependent chronic kidney disease patients. *Advances in Chronic Kidney Disease*.

[271] Kwack C., Balakrishnan V. S. (2006). Managing erythropoietin hyporesponsiveness. *Seminars in Dialysis*.

[272] Vos F. E., Schollum J. B., Coulter C. V., Doyle T. C. A., Duffull S. B., Walker R. J. (2011). Red blood cell survival in long-term dialysis patients. *American Journal of Kidney Diseases*.

[273] Antonelou M. H., Kriebardis A. G., Velentzas A. D., Kokkalis A. C., Georgakopoulou S.-C., Papassideri I. S. (2011). Oxidative stress-associated shape transformation and membrane proteome remodeling in erythrocytes of end stage renal disease patients on hemodialysis. *Journal of Proteomics*.

[274] Zhu Q., Sun Z., Jiang Y., Chen F., Wang M. (2011). Acrolein scavengers: reactivity, mechanism and impact on health. *Molecular Nutrition and Food Research*.

[275] Facorro G., Aguirre F., Florentin L. (1997). Oxidative stress and membrane fluidity in erythrocytes from patients with hemolytic uremic syndrome. *Acta Physiologica, Pharmacologica et Therapeutica Latinoamericana*.

[276] Gomez S. A., Abrey-Recalde M. J., Panek C. A. (2013). The oxidative stress induced *in vivo* by Shiga toxin-2 contributes to the pathogenicity of haemolytic uraemic syndrome. *Clinical & Experimental Immunology*.

[277] Galley H. F. (2011). Oxidative stress and mitochondrial dysfunction in sepsis. *British Journal of Anaesthesia*.

[278] Gitlin J. D. (2003). Wilson Disease. *Gastroenterology*.

[279] Attri S., Sharma N., Jahagirdar S., Thapa B. R., Prasad R. (2006). Erythrocyte metabolism and antioxidant status of patients with wilson disease with hemolytic anemia. *Pediatric Research*.

[280] Nagasaka H., Inoue I., Inui A. (2006). Relationship between oxidative stress and antioxidant systems in the liver of patients with Wilson disease: hepatic manifestation in wilson disease as a consequence of augmented oxidative stress. *Pediatric Research*.

[281] Lang P. A., Kasinathan R. S., Brand V. B. (2009). Accelerated clearance of *Plasmodium*-infected erythrocytes in sickle cell trait and annexin-A7 deficiency. *Cellular Physiology and Biochemistry*.

[282] Basu S., Banerjee D., Chandra S., Chakrabarti A. (2008). Loss of phospholipid membrane asymmetry and sialylated glycoconjugates from erythrocyte surface in haemoglobin E *β*-thalassaemia. *British Journal of Haematology*.

[283] Haynes J., Obiako B., King J. A., Hester R. B., Ofori-Acquah S. (2006). Activated neutrophil-mediated sickle red blood cell adhesion to lung vascular endothelium: role of phosphatidylserine-exposed sickle red blood cells. *American Journal of Physiology—Heart and Circulatory Physiology*.

[284] Kaul D. K., Tsai H. M., Liu X. D., Nakada M. T., Nagel R. L., Coller B. S. (2000). Monoclonal antibodies to alpha V beta 3 (7E3 and LM609) inhibit sickle red blood cell-endothelium interactions induced by platelet-activating factor. *Blood*.

[285] Kumar A., Eckman J. R., Wick T. M. (1996). Inhibition of plasma-mediated adherence of sickle erythrocytes to microvascular endothelium by conformationally constrained RGD-containing peptides. *American Journal of Hematology*.

[286] Setty B. N. Y., Kulkarni S., Stuart M. J. (2002). Role of erythrocyte phosphatidylserine in sickle red cell-endothelial adhesion. *Blood*.

[287] Haynes J., Obiako B., Hester R. B., Baliga B. S., Stevens T. (2008). Hydroxyurea attenuates activated neutrophil-mediated sickle erythrocyte membrane phosphatidylserine exposure and adhesion to pulmonary vascular endothelium. *The American Journal of Physiology—Heart and Circulatory Physiology*.

[288] Hillery C. A., Du M. C., Wang W. C., Scott J. P. (2000). Hydroxyurea therapy decreases the in vitro adhesion of sickle erythrocytes to thrombospondin and laminin. *British Journal of Haematology*.

[289] Setty B. N. Y., Kulkarni S., Dampier C. D., Stuart M. J. (2001). Fetal hemoglobin in sickle cell anemia: relationship to erythrocyte adhesion markers and adhesion. *Blood*.

[290] Yasin Z., Witting S., Palascak M. B., Joiner C. H., Rucknagel D. L., Franco R. S. (2003). Phosphatidylserine externalization in sickle red blood cells: associations with cell age, density, and hemoglobin F. *Blood*.

[291] Charache S., Terrin M. L., Moore R. D. (1995). Effect of hydroxyurea on the frequency of painful crises in sickle-cell-anemia. *The New England Journal of Medicine*.

[292] Kirk K. (2001). Membrane transport in the malaria-infected erythrocyte. *Physiological Reviews*.

[293] Duranton C., Huber S. M., Tanneur V. (2003). Electrophysiological properties of the *Plasmodium falciparum*-induced cation conductance of human erythrocytes. *Cellular Physiology and Biochemistry*.

[294] Brand V. B., Sandu C. D., Duranton C. (2003). Dependence of *Plasmodium falciparum* in vitro growth on the cation permeability of the human host erythrocyte. *Cellular Physiology and Biochemistry*.

[295] Lang F., Lang P. A., Lang K. S. (2004). Channel-induced apoptosis of infected host cells—the case of malaria. *Pflugers Archiv*.

[296] Huber S. M., Duranton C., Lang F. (2005). Patch-clamp analysis of the ‘new permeability pathways’ in malaria-infected erythrocytes. *International Review of Cytology*.

[297] Lew V. L., Tiffert T., Ginsburg H. (2003). Excess hemoglobin digestion and the osmotic stability of *Plasmodium falciparum*-infected red blood cells. *Blood*.

[298] Joshi P., Gupta C. M. (1988). Abnormal membrane phospholipid organization in *Plasmodium falciparum*-infected human erythrocytes. *British Journal of Haematology*.

[299] Schwartz R. S., Olson J. A., Raventos-Suarez C. (1987). Altered plasma membrane phospholipid organization in *Plasmodium falciparum*-infected human erythrocytes. *Blood*.

[300] Ayi K., Giribaldi G., Skorokhod A., Schwarzer E., Prendergast P. T., Arese P. (2002). 16alpha-bromoepiandrosterone, an antimalarial analogue of the hormone dehydroepiandrosterone, enhances phagocytosis of ring stage parasitized erythrocytes: a novel mechanism for antimalarial activity. *Antimicrobial Agents and Chemotherapy*.

[301] Turrini F., Ginsburg H., Bussolino F., Pescarmona G. P., Serra M. V., Arese P. (1992). Phagocytosis of *Plasmodium falciparum*-infected human red blood cells by human monocytes: involvement of immune and nonimmune determinants and dependence on parasite developmental stage. *Blood*.

[302] Sherman I. W., Eda S., Winograd E. (2004). Erythrocyte aging and malaria. *Cellular and Molecular Biology*.

[303] Sherman I. W., Eda S., Winograd E. (2003). Cytoadherence and sequestration in *Plasmodium falciparum*: defining the ties that bind. *Microbes and Infection*.

[304] Koka S., Föller M., Lamprecht G. (2007). Iron deficiency influences the course of malaria in *Plasmodium berghei* infected mice. *Biochemical and Biophysical Research Communications*.

[305] Koka S., Huber S. M., Boini K. M., Lang C., Föller M., Lang F. (2007). Lead decreases parasitemia and enhances survival of *Plasmodium berghei*-infected mice. *Biochemical and Biophysical Research Communications*.

